# 

**Published:** 1803-03-01

**Authors:** 


### TO CORRESPONDENTS.

The Editors request the author of the proposal for a radical cure of bubonocele, signed S. C. and printed in our last number, p. 161, &c. to transmit his name; otherwise, they shall be under the necessity of giving the priority of that proposal to Mr. Carlisle, who publicly signified his intention to perform such an operation at the weekly consultation of the Westminster Hospital seven weeks before the receipt of S. C.'s letter.

We have received observations on that proposal; but as it is not yet in an authentic form before the public, we have postponed their appearance for the present.

Mr. Nunn's letter, requesting that his case might be returned, as farther investigation had convinced him that it was erroneously stated, did not arrive till after it was printed, and too late to be altered: We therefore request our readers to pay no attention to the statement, till it is properly corrected.

Communications are received from Dr. Noehden, and Dr. Walker, but too late and too long for insertion at present; from Mr. Reece, Mr. Wagstaffe, Mr. Dennet, Mr. Washbourn, P. Q. and from Mr. Jardine by Mr. Blair.

